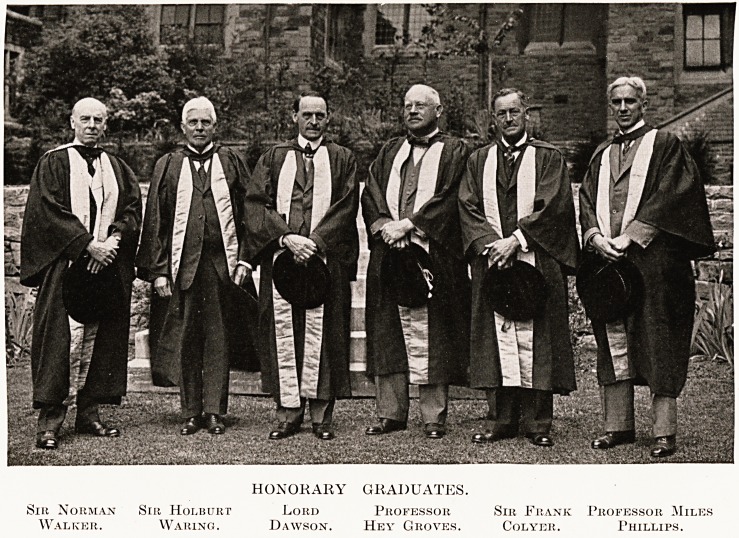# The Centenary of the Foundation of the Bristol Medical School

**Published:** 1933

**Authors:** 


E. FAWCETT, M.D., F.R.S.
Professor of Anatomy, 1893.
Dean of Faculty of Medicine, 1905.

### THE CENTENARY OF THE FOUNDATION OF THE BRISTOL MEDICAL SCHOOL.

---

THE centenary of the Bristol Medical School was celebrated by the University of Bristol on 30th June and 1st July, 1933. A history of the School, written for this occasion by Dr. George Parker, relates its origin from the fusion of several private schools of anatomy, of which the first was started in 1744, seven years after the opening of the Bristol Infirmary. In 1815 the Society of Apothecaries of London was authorized by Act of Parliament to examine and license all new apothecaries, as the general practitioners were called at that time, and to prosecute unqualified apothecaries. This Act required that candidates for qualification should attend at proper lectures and clinics. The Apothecaries' Society gave "recognition" to some of the lecturers in Bristol as early as 1828, and by

1831 a medical student could attend the whole course in medicine at Bristol, and obtain the licence of the Society of Apothecaries without any period of residence in London. This attitude of the Apothecaries' Society made provincial medical schools possible in England. The Royal College of Surgeons strenuously opposed the provincial schools, insisting on attendance at a hospital of 100 beds in London or in certain Scottish or Irish medical centres.

Bristol formed in 1833 a very strong school by the combination of lecturers from various groups, urged thereto by Dr. Carrick. He was a Scottish M.D., senior physician at the Infirmary, and a man of great foresight and character. Carrick delivered the inaugural address at the opening of the School on 14th October, 1833. In this address he observed that now for the first time instruction could be given in Bristol in every department of medical science, and he expressed the hope that the medical school would before long form an integral part of the then flourishing "Bristol College," and share in its general literary and scientific training. Soon all the rival schools disappeared, and in 1834 the College of Surgeons was forced to capitulate, offering the same recognition as the Apothecaries had done. Thus, the Bristol Medical School was launched on a course which led by various stages to incorporation in the University College (founded in 1876). Ultimately, when the University of Bristol received its Charter in 1909, the old Medical School became the Faculty of Medicine in the University. It is this unbroken existence during one hundred years that has just been celebrated. The Dean, Professor E. Fawcett, M.D., F.R.S., has occupied the Chair of Anatomy during forty years of that century, and has been Dean of the Medical Faculty since 1905—a record of valued service and unremitting energy that it will be hard to equal.[1]

On Friday, 30th June, 1933, the past and present members and students of the Bristol Medical School, delegates from sister universities and the ancient Medical Corporations and guests were entertained at garden parties, given by the respective Presidents, at the Royal Infirmary and the General Hospital. In the evening they assembled to the number of 250 at the University Union to what Pepys would have called "a sorry, poor dinner."

The Dean of the Faculty of Medicine, Professor Edward Fawcett, F.R.S., was in the chair. In addition to Delegates and Honorary Graduands there were present the Lord Mayor of Bristol, the Lady Mayoress, the Sheriff (Dr. Kenneth Wills) and Mrs. Wills.

After dinner the Vice-Chancellor received members, delegates and guests to the number of 1,200 at the University. A feature of the evening was an exhibit of Library incunabula and objects of interest connected with the School: among other exhibits were a collection of the various medals offered for competition in the School, rare and exceptionally interesting publications by bygone Bristol medical men, and curiosities, such as the stocking of Cotter, the Irish giant.

### CONGREGATION FOR CONFERRING HONORARY DEGREES.

The central feature of the official celebrations was the Congregation for conferring Honorary Degrees, held in the Hall of the University on the morning of Saturday, July 1st. Public expectation had been roused by a rumour that the Chancellor would on this

[1] Reprinted, by courtesy of the Editor, from the *British Medical Journal*, 1933, vol. ii., p. 70.

unique occasion break through the rule that he has laid upon himself since his installation, and actually appear in person to crown the ceremonies and himself to confer the degrees ; but this hope proved illusory.

The Vice-Chancellor presided over the congregation.

Present :—Pro-Chancellors Dr. S. H. Badock, Dr. H. C. Baker ; Pro-Vice-Chancellor Professor J. F. Dobson.

Delegates from Universities : Professor E. Bramwell, F.R.C.P. (Edinburgh), Professor W. Langdon Brown, F.R.C.P. (Cambridge), Professor A. H. Burgess, F.R.C.S. (Manchester), Sir E. Farquhar Buzzard, Bt., K.C.V.O., F.R.C.P. (Oxford), Mr. P. T. Crymble, F.R.C.S. (Belfast), Dr. W. R. Dawson (Dublin), Professor W. J. Dilling (Liverpool), Sir Ernest Graham Little, M.P., F.R.C.P. (London), Professor A. Hunter, F.R.S.E. (Glasgow), Professor Stuart McDonald, F.R.C.P. (Durham), Professor J. W. McLeod (Leeds), Professor G. P. McLoughlin (Ireland), Professor L. G. Parsons, F.R.C.P. (Birmingham), Professor Miles H. Phillips, F.R.C.S. (Sheffield), Professor A. W. Sheen, C.B.E., F.R.C.S. (Wales), Professor T. Shennan, F.R.C.S. (Aberdeen).

In place of a delegate, the University of St. Andrews sent an address of congratulation to the Bristol Medical School.

Representatives of Medical Corporations : The Right Honourable Lord Dawson of Penn, P.C., G.C.V.O., K.C.B. (Royal College of Physicians of London), Sir Holburt J. Waring, P.R.C.S. (Royal College of Surgeons of England), Dr. R. C. B. Wall, F.R.C.P., M.S.A. (Society of Apothecaries), Sir Norman Walker, P.R.C.P. (President, General Medical Council).

The Vice-Chancellor declared the Congregation open for the conferment of degrees.

### Professor Miles Harris Phillips, F.R.C.S.

The Public Orator: "The first whom I am to present is one who began his studies in this place, and whom we may therefore claim in some sense as a distinguished alumnus of our own. Professor Miles Harris Phillips was a student in the University College under our present Dean of Medicine, and completed his studies at King's College, London, where he took honours in obstetric medicine. Since then his important work as Surgeon to the Jessop Hospital in Sheffield and as Professor of Obstetrics and Gynæcology in the University there has won him a position of distinction and authority in the branch of therapeutics which he has made his own. Among other important services I may mention that he served for four years upon the Departmental Committee set up by the Ministry of Health to study the problem of Maternal Mortality, and undertook special investigations into the midwifery practice and methods of teaching Clinical Obstetrics in Holland, Denmark and Sweden.

"I present to you, therefore, Professor Miles Harris Phillips as eminently worthy of the degree of Doctor of Medicine, *honoris causa.*"

### Professor Ernest William Hey Groves, M.D., F.R.C.S.

"The next case, Mr. Vice-Chancellor, is also drawn from our neighbourhood, but the symptoms are different. Mr. Hey Groves's eminence in the practice and teaching of Orthopædic Surgery, and especially in that branch of it which deals with diseases and fractures of bone, is very well known to you. He was for twenty-nine years on the staff of the Bristol General Hospital, and during that period gained many of the awards and honours which are reserved for men in the first rank of his profession. He has been Jacksonian Prizeman, Bradshaw Lecturer, Hunterian Orator, and he has held the Long Fox Lecturership in this University. He has been a Member of Council of the Royal College of Surgeons since 1918, and in 1929 was elected Vice-President. In 1930 he became President of the British Orthopædic Association, and in 1931 President of the Association of Surgeons of Great Britain and Ireland.

"It is a privilege for me to present as a colleague a man so distinguished in a profession where the standard of distinction is so high. His tenure of the Chair of Surgery in this University came to an end last year; but we are glad to think that as Professor Emeritus he is still a member of our body, and that we may still depend upon his friendly counsel and goodwill.

"I present to you Professor Emeritus Ernest William Hey Groves as eminently worthy of the degree of Doctor of Science, *honoris causa*."

**Sir Frank Colyer, K.B.E., F.R.C.S.**

"Mr. Vice-Chancellor.

"There is no subject for which the ordinary man has a more profound and anxious respect than Dental Pathology, and none which does more to sharpen his memory of the Greek he learned at School: πάσχω, I suffer; πείσομαι, I shall suffer; ἔπαθον, I did suffer; —why didn't I have gas?

"But it is not only, or even chiefly, as an upholder of classical studies that I present Sir Frank Colyer, but as an eminent authority in a department of science which is found to be of great and increasing importance in every sphere of curative medicine.

"As surgeon, for over thirty years, to the Royal Dental Hospital he has been insistent in demanding of his students a sound knowledge of general pathology, and his teaching and example have had far-reaching effects upon dental practice in this country.

"During the War he rendered distinguished services as Consulting Dentist Surgeon to the Croydon War Hospital; and it was in recognition of these services especially that His Majesty was pleased to confer upon him the honour of knighthood.

"He is the author of a standard book on Dental Surgery, and other works on odontological subjects, especially on the variations and diseases of the teeth of animals. But the work which, I believe, has given him special pleasure, and legitimate pride, is that which he has done for the Odontological Collection of the Royal College of Surgeons. He has been Honorary Curator of this collection for thirty years, and has had the honour of seeing a prize founded by the Royal College of Surgeons in his name.

"I present to you, therefore, Frank Colyer, Knight, as eminently worthy of the degree of Doctor of Laws, *honoris causa.*"

### Sir Norman Walker, M.D., P.R.C.P.

"Mr. Vice-Chancellor.

"There is no department of science which has done more to relieve human misery than Dermatology, and it is in this special field that Sir Norman Walker is an eminent English authority. I say "English" for the sake of euphony and in a spirit of patriotism; but I shall have to admit, if challenged, that like so many good things and men he comes from Scotland.

"Graduating at Edinburgh, he early devoted himself to dermatological research, and after studying in the United States, in Prague, and in Hamburg (where he was a pupil of Unna) he was appointed Assistant to the Skin Department of the Royal Infirmary in Edinburgh at the early age of twenty-nine. He was appointed Physician there in 1906, and in the same year was made University Lecturer in Dermatology, a post which he held until 1924. His reputation as a teacher of his subject drew students to Edinburgh from all over the world, and his pupils are to be found now in every country. Of his literary works two were published in Bristol, a translation of Hansen's *Leprosy* and an *Introduction to Dermatology*. He is also the translator of the monumental work by Unna on the *Histopathology of the Skin*. Since 1906 he has represented the Medical Practitioners of Scotland on the General Medical Council; has served as Chairman of the Business Committee, and of the Examination Committee; and since 1931, when Sir Donald Macalister retired, has held the very onerous and responsible post of President. In the service of medical education outside this country he has paid two visits to India at the request of successive Secretaries of State to report on medical education in that country, and two visits to the United States to discuss means of closer co-operation in medical studies between the two nations. He is a Member of the Court of Edinburgh University and Chairman of the Consultative Council for Medical and Allied Services under the Scottish Department of Health.

"I therefore present Norman Walker, Knight, as eminently worthy of the degree of Doctor of Laws, *honoris causa*."

HONORARY GRADUATES.

Sir Norman Walker. Sir Holburt Waring. Lord Dawson. Professor Hey Groves. Sir Frank Colyer. Professor Miles Phillips.

### Sir Holburt Jacob Waring, P.R.C.S.

"Mr. Vice-Chancellor,

"I have already had the privilege of presenting to you the Vice-President of the Royal College of Surgeons. I am now to present the President, Sir Holburt Jacob Waring, a man distinguished both for his work as a surgeon and for his many and eminent services in the cause of medical education. Since 1902, when he was appointed Assistant Surgeon, to 1931, when he completed twenty-two years' service as Surgeon, he has been a member of the staff of St. Bartholomew's Hospital, and during that time has established a great reputation and has won many professional honours. Among these I may mention his appointment as Bradshaw Lecturer in 1921 and as Hunterian Orator in 1928. In the academic sphere his services have been equally conspicuous. He has been Dean of Medicine and Examining Surgeon in the University of London, and from 1922 to 1924 held the office of Vice-Chancellor. He has performed the duties of Treasurer to the General Medical Council (of which he was for many years a valued member) and of Treasurer to the Dental Board and to the London School of Hygiene and Tropical Medicine. He is the author of important works on surgery, and in particular of a standard work on *Diseases of the Liver and Gall-bladder and Biliary System.*

"His authority in this domain, and the confidence that is reposed in his judgement in all matters affecting these sensitive and delicate regions is established conclusively by the fact that the General Medical Council — that very prudent body — appointed Sir Holburt Waring to the most intimate and momentous post of which they have to dispose: I refer to the post of Cellarer.

"It is therefore with great confidence, Mr. Vice-Chancellor, that I present Holburt Jacob Waring, Knight, as eminently worthy of the degree of Doctor of Laws, *honoris causa.*"

**The Right Honourable Lord Dawson of Penn, P.C., G.C.V.O., K.C.B., LL.D., D.C.L., P.R.C.P., F.R.C.S.**

"Mr. Vice-Chancellor,

"Last of our distinguished graduands I present to you one who has already been honoured by many Universities for his services to medical education and his contributions to medical literature.

"Throughout his career Lord Dawson of Penn has been attached to the London Hospital, and has taken an active part in the clinical teaching of the Medical School belonging to that institution. In the academic sphere he has been Dean of the Faculty of Medicine in the University of London, and is a Member of the Senate. He is a member of the Medical Research Council, and since 1931 has been President of The Royal College of Physicians. This year he is also President of the British Medical Association. Of his many important contributions to medical literature I shall not attempt, as a layman, to give you any account. But I shall be content simply to give the title of one, because it offers a challenge to a conscientious public orator, and I want to see if I can do it. It is entitled *Spirochœtosis ictero-hœmorrhagica*—and that, Mr. Vice-Chancellor, is, I believe, merely a handy title used for convenient reference. During the four years of the European War he served as Physician Consultant in France, and at the conclusion of hostilities was made

Chairman of the Council set up by the Government to devise a scheme of hospital co-ordination and health services throughout the country.

"His Majesty was pleased to ennoble him in 1920, and he took his seat in the House of Lords as Baron Dawson of Penn. His interventions in debate have been confined to scientific and social subjects, in which his great knowledge and experience have given his opinion authoritative weight. He has had the honour to be appointed Physician-Extraordinary to his late Majesty King Edward, and Physician-in-Ordinary to H.M. King George and H.R.H. the Prince of Wales.

"Such, in brief and inadequate outline, is this record of his honours and achievements. And I might end here. But in presenting the last of these eminent men I should like to impose upon him the burden and the duties of a symbol. I should like to say that as President of the Royal College of Physicians Lord Dawson worthily embodies, in his office as in his person, that great tradition of integrity, of devotion, of fearless surmise and faithful question which has built up the great art of healing, and to which, in its varied manifestations and powers we have endeavoured to do honour to-day.

"I present to you the Right Hon. Bertrand, Lord Dawson of Penn, as eminently worthy of the degree of Doctor of Laws, *honoris causa.*"

LUNCHEON AT THE UNIVERSITY UNION.

The Vice-Chancellor presided and proposed the toast of the Honorary Graduates.

Lord Dawson of Penn, in reply, said:—

"It is indeed a high honour to be associated with the Bristol Medical School on this historic occasion. For centuries Bristol proudly claimed to be the foremost provincial city and port of the realm; and it is therefore natural when we turn our eyes to the past to think first of Bristol as a port. One vessel in particular that sailed from this port is of interest to medical men, for in it sailed as an officer Dr. Dover, now remembered chiefly as the inventor of the powder that bears his name. This vessel, one of the first to circumnavigate the globe, brought back to Bristol Alexander Selkirk from his lonely exile; and it was on the quays of Bristol that he met Daniel Defoe, who immortalized him as Robinson Crusoe.

"Bristol men indeed have always been famed for their enterprise and adventure. That that enterprise has persisted is witnessed by the great progress of your University. The Bristol Medical School of which your University is the child—or possibly grandchild—was fortunate in the early days of its history in finding leaders of thought and action: such men as Budd, Beddoe, W. B. Carpenter, J. G. Swayne, and Symonds. But illustrious as these were, the Faculty of Medicine need fear no comparison of the present with the past. And no small part of the position which your school occupies to-day is due to Professor Fawcett. It is seldom indeed that in any University there has been such a record as that of your Dean—for forty years Professor of Anatomy, and for twenty-eight Dean of the Faculty of Medicine. It is to us an honour to pay him homage, a homage that is enshrined in our affections.

"I have been interested recently in the subject of Honorary Degrees. And among other curious discoveries I encountered the Degree of Master in Grammar. It was bestowed upon those schoolmasters whom it was desired to honour with a special distinction, and the accompanying ceremonies throw an interesting sidelight on the educational methods of a bygone age. The new Graduate was accosted by a Bedell, who presented a rod, with which the Master forthwith proceeded to flog a boy: the Bedell and the boy each received a groat for their 'pains.'

"I think it is true that medical education is at the parting of the ways.[1] The reason is this: Medicine has so grown in scope that there is now serious danger of the branches of the tree overweighting the trunk.

"As a result of the increase of the branches of medicine, the curriculum is so overweighted that there is a serious danger that we shall not produce the medical men of the quality we desire.

"It is part of the duty of all medical faculties throughout the country to address themselves to the problem of how the curriculum can be altered in order to bring about the prime object of all education. That is, first, to train the mind, and, secondly, to instil into the students the basic facts that must make the foundation of any well-educated medical man.

"That foundation at the present moment is being imperilled for the reason that each year that goes by there comes along a new subject, and that new subject is heaped on the already full curriculum.

"It would be adjusted, no doubt, by making the branches of the profession take their proper place as skilled crafts that are learned subsequently to the basic education required for the qualification of the student.

"It is true, craftsmen in this or that subject are needed. Not only are they needed, but they need to be more skilled as the years go by. But be it remembered that specialized crafts are good

[1] The conclusion of Lord Dawson's speech is reproduced by courtesy of the Editor from the *Western Daily Press*, 3rd July.

servants, but bad masters. They need to be controlled and guided by what we may call the wisdom of medicine which belongs to the trunk, and not to the branches of the tree."

Acknowledging the tribute of Lord Dawson, Professor Fawcett said that few men indeed were so fortunate as he had been to enjoy the privilege of serving the University through forty years. And particularly, that he had been able to take his part, as Dean of the Faculty of Medicine, in the celebration of the Centenary of the Medical School. He sincerely thanked all those who had worked so hard to make those celebrations a success.

There remained unfulfilled one of his long-cherished ambitions: to see established at the University of Bristol a scholarship to be bestowed on the most distinguished graduate of the year. Certain gifts had been made or promised, and he was hopeful that more would be forthcoming, as centenary gifts to the Medical School, towards the establishment of such scholarship; and when that had been achieved he, at least, would feel that his long connection with the School had been crowned with success.

The centenary celebrations concluded with a garden party given by the Sheriff and Mrs. Wills in the garden of the Royal Fort.

On July 29th a deputation visited University College, London, by kind permission of the Provost, to confer the degree of Doctor of Science on Professor Grafton Elliot Smith, LL.D., F.R.C.P., who was unfortunately unable to be present at the centenary celebrations. The Vice-Chancellor presided.

Present: Pro-Vice-Chancellor Professor J. F. Dobson; Dean of the Faculty of Medicine Professor

Edward Fawcett, F.R.S.; Dean of the Faculty of Science Professor J. E. Lennard Jones, D.Sc.

The Public Orator:

"The science of comparative anatomy, and particularly the comparative anatomy of the brain, has had a profound effect upon modern theories of evolution, and so upon anthropology and the historical study of early civilizations. It is in this wide field of science and philosophy that Professor Elliot Smith is one of the chief living authorities.

"It was while helping archæologists in Egypt to interpret the anatomical evidence provided by human remains that he became interested in archaeology, and was able to invest his already extensive researches into the mechanism of the brain with a wider and more philosophical import. Of his researches it is not for a layman to speak; but those who are competent to judge would probably regard his work on the casts of the interior of the skull, and his more recent researches into stereoscopic vision as a factor in the evolution of intelligence, as contributions of outstanding importance.

"I may mention too that he first determined the function of a certain minute organ or accessory of the brain which is called the 'Hippocampus,' or 'sea-horse.'

"Yes, Mr. Vice-Chancellor, it appears that we all have a sea-horse on the brain. And what the moral or political significance of that may be, I feel that in the absence of our Chancellor we must not venture to speculate. But it is reassuring to know that Professor Elliot Smith has put a hook in its nostrils, and that the solution to all our anxieties in this matter is to be found in his book.

"He is the author of a series of works on evolution and early culture, which while addressed primarily to specialists have by their charm of style and skill of exposition gained a wide circle of non-professional readers. The Muses have never been banished from his laboratory, and in his hands the grimmest anthropoid skull has yielded Minerva in full arms. At Manchester, where he formerly held the Chair of Anatomy, he was President of the Literary and Philosophical Society. He is now Professor of Anatomy in the University of London, and has been President of the Anatomical Society of Great Britain and Ireland.

"I present to you Professor Grafton Elliot Smith as eminently worthy of the degree of Doctor of Science, *honoris causa*."

## Figures and Tables

**Figure f1:**
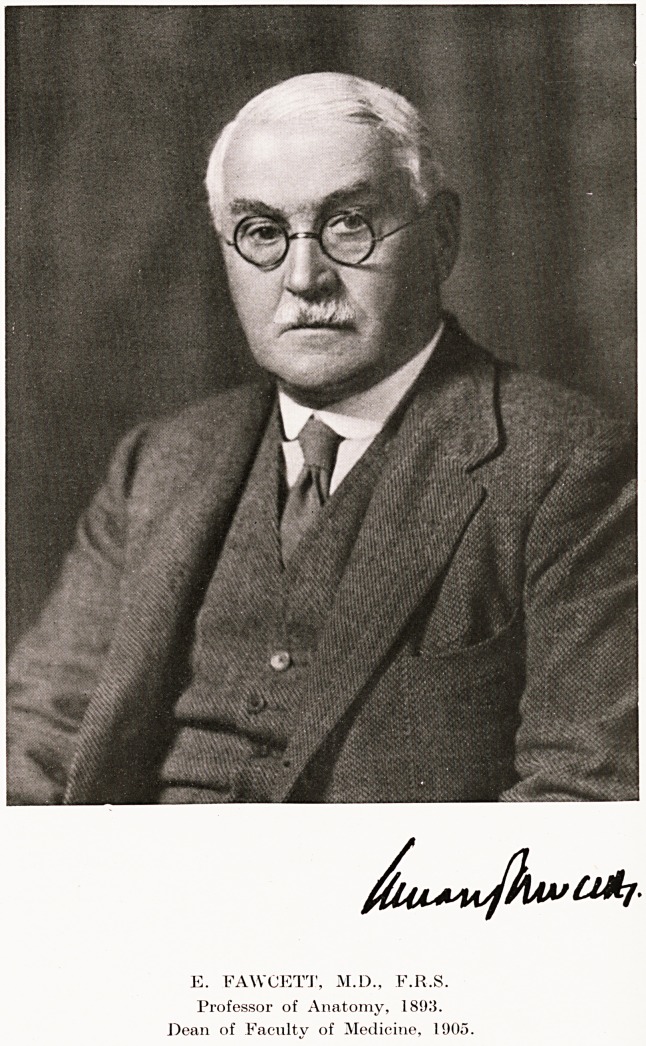


**Figure f2:**